# Markers of Inflammation and Vascular Parameters in Selective Progesterone Receptor Modulator (Ulipristal Acetate)-Treated Uterine Fibroids

**DOI:** 10.3390/jcm10163721

**Published:** 2021-08-21

**Authors:** Iwona Szydłowska, Marta Grabowska, Jolanta Nawrocka-Rutkowska, Andrzej Kram, Małgorzata Piasecka, Andrzej Starczewski

**Affiliations:** 1 Department of Gynecology, Endocrinology and Gynecological Oncology, Pomeranian Medical University in Szczecin, Unii Lubelskiej 1 Street, 71-252 Szczecin, Poland; jolanaw@poczta.onet.pl (J.N.-R.); andrzejstarcz@tlen.pl (A.S.); 2 Department of Histology and Developmental Biology, Pomeranian Medical University, Żołnierska 48 Street, 71-210 Szczecin, Poland; mpiasecka@ipartner.com.pl; 3 Department of Pathology, West Pomeranian Oncology Center, Strzałowska 22 Street, 71-730 Szczecin, Poland; akram@onkologia.szczecin.pl

**Keywords:** uterine myoma, ulipristal acetate, transforming growth factor β, tumor necrosis factor α, interleukin 6, interleukin 10, mast cells, macrophages, CD31

## Abstract

The exact mechanism of selective progesterone receptor modulator action in leiomyoma still challenges researchers. The aim of the study was to assess the effects of ulipristal acetate (UPA) on immunoexpression of inflammatory markers and vascularization in fibroids. UPA-treated patients were divided into three groups: (1) good response (≥25% reduction in volume of fibroid), (2) weak response (insignificant volume reduction), (3) and no response to treatment (no decrease or increase in fibroid volume). The percentage of TGFβ, IL6, IL10, CD117, and CD68-positive cells were significantly lower in the group with a good response to treatment vs. the control group. Moreover, the percentage of IL10 and CD68-positive cells in the group with a good response to treatment were also significantly lower compared to the no response group. Additionally, a significant decrease in the percentage of IL10-positive cells was found in the good response group vs. the weak response group. There were no statistical differences in the percentage of TNFα-positive cells and vessel parameters between all compared groups. The results of the study indicate that a good response to UPA treatment may be associated with a decrease of inflammatory markers, but it does not influence myoma vascularization.

## 1. Introduction

Uterine fibroids (UFs) are the most common benign tumors of the female reproductive tract. They occur in approximately 70% of women up to 50 years of age. About 30% of myoma is symptomatic. The symptoms include severe uterine bleeding, pelvic pain, uterine incontinence, constipation, and pain during intercourse. In patients at the reproductive age, UFs may cause miscarriages or infertility. These monoclonal tumors are formed in smooth-muscle cells due to a variety of reasons. At this point, it is worth mentioning that, although the majority of existing literature sources consider UFs monoclonal in nature, most recent studies point towards the possibility of leiomyoma as being heterogenous and consisting of diverse and novel cell types (single cell RNA sequencing analysis in MED12 (+) and MED12 (−) variants of leiomyomas) [1]. A combination of severe hypoxia and gonadal steroids stimulates the local expression of the angiogenic growth factor in fibroid tissue, as well as the production of cytokines and chemokines. This in turn impacts cell proliferation and extracellular matrix (ECM) deposition, thus providing a vascular support for growing leiomyoma [2]. Genetic factors may also be associated with the uterine fibroid formation. Cytogenetic assays confirm chromosomal abnormalities in about 40% of myomas. The HMGA2 (high mobility group AT-hook 2) gene is overexpressed in 65% of these types of tumors and mutations in a transcriptional regulator complex subunit 12 (MED12) are demonstrated in 70% of fibroids [3,4]. The exact role of inflammation in UF pathogenesis is a subject of an ongoing debate. It is suggested that local and general inflammatory status may present as possible mechanisms underlying the onset of myomas [3,5,6]. For the purpose of this study, it is assumed that UFs are partly induced by the presence of chronic inflammation and the inflammatory microenvironment in areas of tumor formation. The chronic inflammatory state increases levels of estrogen. Estrogen, binding with estrogen receptors, increases progesterone receptor expression (PR-A and PR-B) and strengthens the effects of progesterone (enhancement of mitotic activity) in the growth of uterine fibroids [7,8]. Other mechanisms, such as the regulation of growth factor function, ECM accumulation, and microRNA (miRNA) expression, are thought to be associated with P4 activity [8,9,10,11]. While progesterone, influenced by growth factors, i.e., transforming growth factor β (TGFβ), increases proliferation and decreases apoptosis in fibroid tissue, the very same growth factors are ‘reliant’ on progesterone itself. Chronic inflammation is sustained by specific cytokines secreted by both immune and tumor cells.

Extracellular matrix deposition, cell proliferation, and angiogenesis are key cellular events implicated in leiomyoma growth. It has been suggested that, over time, fibroids go through natural changes related to collagen accumulation, which results in decreased microvessel density, followed by myocyte injury, atrophy, and eventual senescence and involution due to ischemic cellular degeneration and inanition. Areas of excessive accumulation of collagen are hyalinized and hypocellular. In large tumors, a collagenous ECM is often abundant [12]. The accumulation of ECM in fibroids indicates the inflammatory nature of these tumors and, as such, is a response to the activity of cytokines and growth factors. Inflammatory cells, i.e., monocytes and macrophages, cause, in response to inflammatory signals, typical myoma tumors’ fibrosis. ECM act as a sort of reservoir for growth factors, increasing, stabilizing, and extending their influence. Among these, isoforms of TGF-β are some of the most important ones [13,14]. Major cytokines, namely the expression of interleukin 1 (IL1), 6 (IL6), 11 (IL11), 13 (IL13), 15 (IL15), 33 (IL33), tumor necrosis factor α (TNFα), and the granulocyte-macrophage colony-stimulating factor, have been implicated to leiomyoma pathophysiology. It may therefore be interesting to gain a more thorough understanding of the modulation of the inflammatory process in UFs treated with drugs commonly used in myoma therapy. Previous studies suggest a modulatory mechanism of gonadotropin-releasing hormone analogues (GnRHa) [15] but there is little information on selective progesterone receptor modulator (SPRM) action in this context. Among SPRMs, mifepristone and ulipristal acetate are considered especially efficacious in the treatment of uterine fibroids (reduction in symptoms and volume shrinkage). UPA modulates the PR activity. The drug has different properties (agonistic or antagonistic) in different kinds of tissue. In uterine fibroids, after binding with progesterone receptors, UPA has a PR antagonistic action, which is mediated by the recruitment of corepressors that prevent the transcription of target genes and then by coregulators, leading to non-genomic signaling in the cytoplasm [16]. From the clinical point of view, UPA efficacy has been confirmed in many studies [17,18,19,20]. Some SPRM drugs, namely asoprisnil, vilaprisan, and telapristone, are no longer studied as the possibility of associated severe side effects is too strong [21].

It is a well-established fact that angiogenesis constitutes an important element in the pathological process, including primary tumor growth, invasion, and metastases, as observed in malignant tumors [22]. Angiogenesis is also considered to be equally necessary for the growth and survival of benign fibroid tumors [2]. Another well-established fact is that multiple growth factors involved in angiogenesis are differently expressed in leiomyoma and myometrium. Xu et al. demonstrated that physiological levels of progesterone increased the vascular endothelial growth factor (VEGF) and adrenomedullin (ADM) in myoma cells, and hence promoted angiogenesis in uterine leiomyomas and tumor growth [23]. A well-vascularized capsule surrounding a tumor mass is typical in fibroids [24].

Most studies report the increased expression of angiogenic factors in leiomyoma. TGFβ and its isoforms, alongside other factors such as TNFα, VEGF, and the basic fibroblast growth factor (bFGF), appear to be highly engaged in the pathophysiology of UFs. The involvement of TNFα in leiomyoma cells’ differentiation and later proliferation has been especially thoroughly described [25,26]. Other growth factors such as the epidermal growth factor (EGF), platelet-derived growth factor (PDGF), activin-A, and ADM also play an important role in fibroid growth [2,27,28]. In this context, substances that target angiogenic growth factors and their receptors to block angiogenesis may constitute a promising approach to leiomyoma therapy.

Our previous research demonstrated antiproliferative and antifibrotic activities of SPRM in uterine fibroids [29]. Recent studies, however, appear to place more emphasis on anti-inflammatory and anti-angiogenic action in uterine fibroid therapy. Treatments such as uterine artery embolization (UAE) and the GnRH agonist (GnRHa) have anti-angiogenic mechanisms [2]. Some studies have highlighted dietary phytochemicals as possible therapies for uterine fibroids with anti-inflammatory, anti-proliferative, anti-fibrotic, and anti-angiogenic effects [27]. Nonetheless, to date, little is known about the influence of SPRM on angiogenic activity in myoma.

The aim of the study was to assess the effects of ulipristal acetate (UPA) on the immunoexpression of inflammatory markers such as TGFβ, TNFα, IL6, IL10, CD117, and CD68, and the vascularization in fibroids. Mediators of inflammation, inflammatory cells, and vascularization were evaluated in myoma tumors according to the response to UPA treatment: good, weak, or no response (volume reduction after treatment). Evaluation of the studied parameters in relation to the in vivo response to treatment extends the knowledge of the mechanism of drug action.

## 2. Materials and Methods

### 2.1. Study Groups and Inclusion/Exclusion Criteria

The study was conducted at the Department of Gynecology, Gynecological Endocrinology, and Oncology and the Department of Histology and Developmental Biology at the Pomeranian Medical University in Szczecin within a period of six years between 2015 and 2021. Prior to the commencement of the study, authors received the approval of the Ethics Committee of The Pomeranian Medical University in Szczecin (KB-0012/94/14). Written informed consent was obtained from all participants of the study prior to inclusion.

In preparation for surgical treatment of myomas, all patients received a 5 mg daily dose of UPA (Esmya; Gedeon Richter Plc., Budapest, Hungary) in the form of a tablet for the period of 3 months. Heavy menstrual bleeding leading to anemia in patients with ultrasound detected uterine myomas constituted an indication for the above treatment. Existing fibroids were ≤10 cm in diameter and classified as type 2; 3; 4; 5 and 2–5 [30] according to the International Federation of Gynecology and Obstetrics (FIGO). Patients were excluded from the study on the basis of: (1) other abnormalities present on gynecological examination; (2) malignant lesions of the uterine cervix in the cytology test (abnormal Pap smear results); (3) previous or ongoing liver dysfunction; (4) abnormal results of Doppler sonography of myomas; and (5) preceding or ongoing hormonal therapy <3 months before the commencement of the study. Ultimately, 34 patients aged 33–55 (mean age 42) were included in the study. Throughout the UPA therapy, patients did not receive any other hormonal drugs. Upon completion of the UPA therapy, a surgery (myomectomy/abdominal, supracervical hysterectomy/laparoscopic, or supracervical hysterectomy) was performed. All patients completed the entire course of therapy and none experienced severe side effects. Based on the clinical assessment of the reduction in myoma volume as a result of the UPA treatment, patients in the study group were divided into three sub-groups:

1. patients who responded well (*n* = 20), in which myoma volume decreased by ≥25% after UPA treatment;
2. patients who responded weakly (*n* = 10), in which myoma volume decreased by <25% after UPA treatment;
3. patients with no response to treatment (*n* = 4), in which no decrease or increase in myoma volume observed after UPA treatment.

Samples of leiomyoma tissue were collected during surgical procedures following UPA therapy. The control group consisted of samples of leiomyoma tissue taken from patients without prior UPA treatment during surgery (myomectomy/abdominal, supracervical hysterectomy/laparoscopic, or supracervical hysterectomy). Specimens were obtained from 30 patients aged 33–51 (mean age 41.8 ± 4.6) in whom menorrhagia leading to anemia was an indication for surgical treatment. Ultrasonography confirmed the presence of myoma tumors in the control group (diameter and classification of fibroids vide: study group criteria). Patients in the control group had not received hormonal treatment within 6 months prior to surgery.

A Voluson imaging with a 5-MHz frequency probe and Color Doppler System was used for all transvaginal sonographic examinations. Pelvic transvaginal ultrasonography was performed by the same ultrasonographer. In both groups, the study group and the control group, 1–4 myomas (ranging from 1.5–8.5 cm in diameter) were found during the ultrasound examination. Fibroid volume was calculated by the formula: length × width × height × 0.526. In cases of more than one fibroid, the mean volume of all myomas was measured. Doppler velocimetric measurements of fibroids and uterine arteries were obtained to exclude potential malignant tumors.

### 2.2. Histological Analysis

In multiple myoma cases, a nodule biopsy was collected from the largest tumor and then analyzed. Each fibroid was histologically assessed post-surgery. Collected myomas were fixed in 4% buffered paraformaldehyde and embedded in paraffin. Next, 3 µm-thin sections were cut and placed on the poly-L-lysine-coated slides.

### 2.3. Immunohistochemistry

Myoma sections were deparaffinized and rehydrated. In order to retrieve antigens, the slides were boiled for 30 min in Target Retrieval Solution Citrate (Dako, Glostrup, Denmark) at pH 6.0 (for TGFβ 1, IL6, and IL10) and in Target Retrieval Solution (Dako, Glostrup, Denmark) at pH 9.0 (for TNFα, CD117, CD68, and CD31). Following this, they were washed in PBS. The endogenous peroxidase was blocked using peroxidase-blocking solution (Dako, Glostrup, Denmark) for 10 min at room temperature. In order to determine the immunoexpression of the specific proteins, the following antibodies were used: (1) mouse monoclonal anti-transforming growth factor β antibody (Abcam, Cambridge, UK), diluted 1:200; (2) mouse polyclonal anti-tumor necrosis factor α antibody (Nordic BioSite, Täby, Sweden), diluted 1:300; (3) rabbit polyclonal anti-interleukin 6 antibody (Nordic BioSite, Täby, Sweden), diluted 1:200; (4) rabbit polyclonal anti-interleukin 10 antibody (Nordic BioSite, Täby, Sweden), diluted 1:200; (5) rabbit polyclonal anti-CD117 antibody (Dako, Glostrup, Denmark), diluted 1:500; (6) mouse monoclonal anti-CD68 antibody (Dako, Glostrup, Denmark), diluted 1:50; and (7) mouse monoclonal anti-CD31 antibody (Dako, Glostrup, Denmark), diluted 1:20. Antibodies were diluted in antibody diluent with background reducing components (Dako, Glostrup, Denmark). The sections were incubated with the primary antibodies in a humid chamber for 30 min. Subsequently, the sections were incubated with a complex containing a secondary antibody conjugated with horseradish peroxidase (Dako, Glostrup, Denmark). Next, diaminobenzidine (Dako, Glostrup, Denmark) was applied. All slides were counterstained with Mayer’s hematoxylin (Sigma-Aldrich Co., St. Louis, MO, USA), dehydrated, and cover-slipped. The slides were examined under a light microscope (Olympus BX 41, Hamburg, Germany). The negative controls for reaction specificity were performed.

### 2.4. Quantitative Computer Image Analysis of Immunoexpression of TGFβ, TNFα, IL6, IL10, CD117, CD68, and CD31

Using the ScanScope AT2 scanner (Leica Microsystems, Wetzlar, Germany), TGFβ 1, TNFα, IL6, IL10, CD117, CD68, and CD31-immunostained myoma sections were scanned at a magnification of 400× (resolution of 0.25 μm/pixel). Obtained digital images of the myomas were analyzed on a computer screen using ImageScope viewer software (Aperio Technologies, Vista, CA, USA).

The quantitative analysis of TGFβ 1, TNFα, IL6, IL10, CD117, and CD68 immunoexpression was performed on slides using the *cytoplasmic v9* and *positive pixel count* algorithm (Aperio Technologies, Vista, CA, USA). Other parameters were set to achieve compliance with the visual assessment of color intensity. The analyzed areas were manually determined. The percentage of positive immunostaining for each marker was determined in high-power fields with an average area of 18.2 mm^2^ (for TGFβ 1), 15.9 mm^2^ (for TNFα), 19.3 mm^2^ (for IL6), 19.3 mm^2^ (for IL10), 20.2 mm^2^ (for CD117), and 20.0 mm^2^ (for CD68) in all patients.

For detailed analysis of the CD31 expression in the myomas, the *microvessel analysis v1* algorithm (Aperio Technologies, Inc., Vista, CA, USA) was used. The microvessel density, mean vessel area, and mean vessel perimeter were calculated. The quantitative data for CD31 was assessed in high-power fields with an average area of 19.4 mm^2^ in all patients.

### 2.5. Statistical Analysis

The results were analyzed using Statistica 13.0 software (StatSoft, Krakow, Poland). The arithmetical means (X), standard deviations (SDs), medians, and minimum and maximum values were calculated for each of the parameters. Obtained values failed normal distribution assumption, therefore the non-parametric Kruskal–Wallis test with Dunn’s multiple comparison test for post hoc analysis were used to assess the differences between the groups. Differences at *p* < 0.05 were considered to be statistically significant.

## 3. Results

### 3.1. Demographic and Baseline Characteristic

In myomas taken from patients in the UPA-treated group with a good response to treatment, a significant decrease in volume was revealed (*p* = 0.039). There was no statistically significant difference in age, number of days passed between the completion of therapy and the surgery, and number of fibroids in all UPA-treated groups (good, weak, or no response) in comparison to the UPA-untreated group (Table 1). In the case of total cell density (number of cells/1 mm^2^), a significant increase was noted (*p* = 0.002) in the group with a good response to UPA treatment vs. the control group.

### 3.2. Immunoexpression of Study Markers

**Figure 1:**
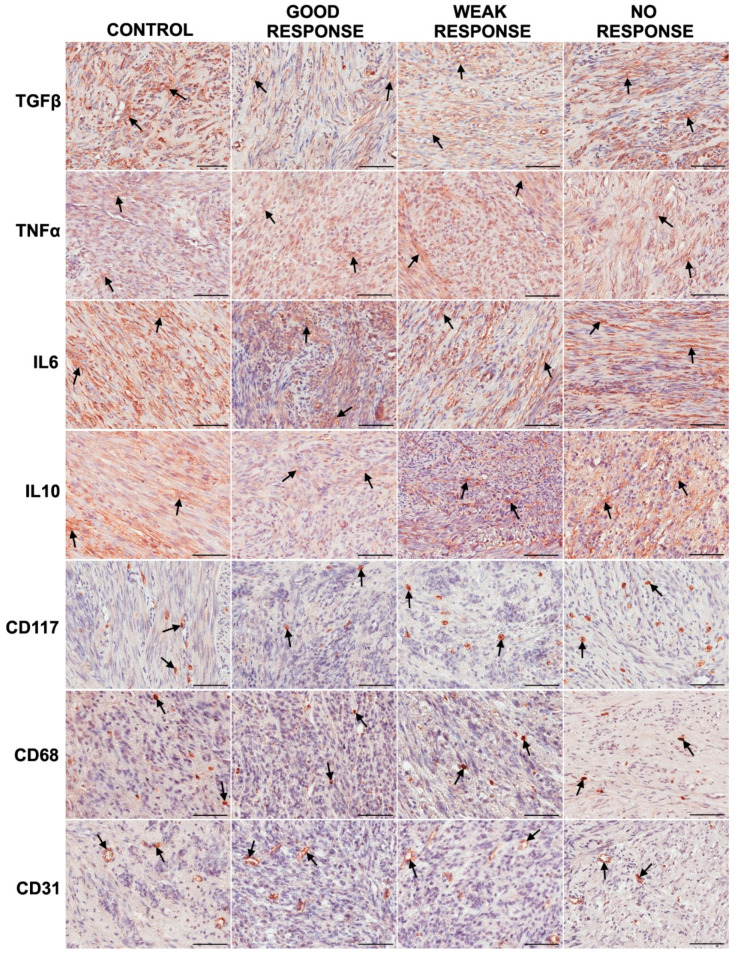
Representative light micrographs of immunoexpression (brown-stained cell cytoplasm indicated by black arrows) of inflammatory markers and vessel marker in fibroid cells in the control group and study groups after 3 months of treatment with ulipristal acetate (UPA): good, weak, and no response to treatment. Note: scale bar—100 µm. Abbreviations: CD31—marker of endothelial cells; CD68—marker of cells of the macrophage lineage; CD117—marker of mast cells; IL6—interleukin 6; IL10—interleukin 10; TGFβ 1—transforming growth factor β 1; and TNFα—tumor necrosis factor α.

Immunoexpression of TGFβ 1, TNFα, IL6, IL10, CD117 (mast cells), CD68 (cells of the macrophage lineage), and CD31 (endothelial cells lining the vessels) in fibroids obtained from the control group and the UPA treated groups was visible in the form of the brown-stained cytoplasm of cells (Figure 1).

### 3.3. Quantitative Analysis of TGFβ and TNFα-Positive Cells

Significant differences were noted only in the percentage of TGFβ 1-positive cells between the control group and the group with a good response to UPA treatment. The percentage of TGFβ 1-positive cells in myomas obtained from groups with weak and no responses to treatment was statistically insignificant in comparison with the UPA-untreated group. The percentage of TGFβ-positive cells in myomas obtained from the UPA-treated group with a good response to treatment was significantly lower (*p* < 0.001) than in the control group. There was no statistical significance in the percentage of TNFα-positive cells between all compared groups (Table 2; Supplementary Figure S1).

### 3.4. Quantitative Analysis of IL6 and IL10-Positive Cells

A significant difference (*p* < 0.001) in the percentage of IL6-positive cells was revealed between the group with a good response to UPA-therapy and the control group. There was no statistical significance in the percentage of IL6 -positive cells in groups with weak or no response to treatment in comparison with the UPA-untreated group. Significant differences (*p* < 0.001 and *p* < 0.05) in the percentage of IL10-positive cells were noted between the group with a good response to UPA therapy, the control group, and groups with weak and no response to treatment (Table 2; Supplementary Figure S1).

### 3.5. Quantitative Analysis of CD117 and CD68-Positive Cells

The analysis of CD117-positive mast cells confirmed that the percentage of these cells was significantly lower (*p* < 0.001) in the group with a good response to UPA treatment vs. the control group. The percentage of CD117-positive mast cells in myomas obtained from the UPA-treated groups with weak or no response to treatment was statistically insignificant in comparison to the untreated group. Significant differences were also observed in percentage of CD68-positive cells between the group with a good response vs. control and no response groups (*p* < 0.001 and *p* < 0.05, respectively). There was no statistical significance in the percentage of CD68-positive cells in the group with a weak response to treatment (Table 2; Supplementary Figure S1).

### 3.6. Quantitative Analysis of CD31 Immunoexpression

The number of vessels per unit area (microvessel density), the mean vessel area, and mean vessel perimeter showed no statistical significance in all compared groups (Table 3).

## 4. Discussion

This study is a continuation of our previous research on the SPRM (UPA) mechanism of action in fibroid tissue [29]. Understanding the mechanism of biological effects of UPA in the treatment of leiomyoma has lately been a subject of much research [29,31,32,33,34,35,36,37,38]. Some studies confirm antiproliferative and antiangiogenic properties of the drug, as well as apoptosis stimulation and a decrease of ECM volume in fibroids; other note differences in the expression patterns of proteins related to cell cycle regulation, remodeling of cell cytoskeleton and drug resistance is influenced by UPA treatment. Patterns of differential response to UPA have also been studied at the genetic and proteolysis level. Recent molecular analysis reveal significant changes in gene expression depending on fibroids’ response to UPA treatment. These findings substantially complement the existing body of knowledge and have been necessary for a general understanding of the UPA mechanism of action. Nonetheless, new mechanisms of the SPRM action are being investigated on ongoing basis in order to further understand the nature of the drug in myoma tissue. We decided to expand the existing information in the context of inflammation and vascularization.

The effect of GnRHa in reducing inflammation and angiogenesis in uterine myoma tissue has been reported in the literature as a possible mechanism for tumor regression. Antigen CD68 is considered to be a marker for activated macrophages [14]. Miura et al. have shown that submucosal and intramural myomas are characterized by higher than normal myometrium and submucosal myoma macrophage infiltration [39]. Khan et al. performed an immunohistochemical analysis of CD68 and observed that the infiltration of CD68-positive cells was significantly decreased in uterine myomas in the GnRHa-treated group. Using antibodies of the von Willebrand factor (vWF) as a vessel marker for angiogenesis, a significantly decreased angiogenic response in GnRHa-treated fibroids was noted [15]. Khan et al. highlighted a stimulatory role of the human inflammatory heat shock protein 70 (HSP70) in leiomyoma growth and observed significantly reduced levels of HSP70 in tissue of GnRHa-treated fibroids [40]. To the best of our knowledge, there is little data on such a response to UPA treatment. This study evaluated predominantly intramural leiomyomas. In comparison to the UPA-untreated group of patients, a significantly decreased infiltration of CD68 was observed in the UPA-treated group of women with a good response to the drug. A weaker decrease in the percentage of CD68-positive cells was noted in groups with low or no response to UPA treatment.

To date, there appears to be little or no studies on the post-SPRM-treatment effect on CD117 in myoma. It is known that mast cells are involved in the induction and pathogenesis of many inflammatory diseases and produce mainly pro-inflammatory cytokines [41]. Mast cells were also found in uterine fibroids [42]. In their study, Nakayama et al. evaluated the number of mast cells in GnRHa-treated and untreated leiomyomas [43]. They observed increased mast cell numbers in the case of leiomyoma weakly responding to GnRH-a therapy, which may explain the mechanism of resistance to GnRHa. Similar effects were observed in our study. Using the CD117 as a marker of mast cells, we have found a significantly lower percentage of CD117-positive mast cells in fibroid tissue in the group with a good response to UPA treatment vs. untreated fibroids. A slightly higher percentage of mast cells was noted when myomas had responded weakly or when there had been no response to UPA treatment.

ECM accumulation is affected by growth factors, cytokines, and steroid hormones. ECM is recognized as a reservoir for growth factors and cytokines [10]. A drug decreasing ECM may also influence degradation of cytokines and growth factors, and thus may regulate tissue organization and angiogenesis that controls leiomyoma growth and associated clinical symptoms. Courtoy et al. [33] demonstrated that a long-term treatment of fibroids with UPA (10 mg) causes an increase in cell density with a decrease in ECM volume fraction. This was not confirmed in the case of a short-term UPA (5 mg) treatment. Numerous studies described leiomyoma cells exhibiting a significantly greater expression of IL1, IL4, IL5, IL6, IL10, IL11, IL13, and IL15 [5,26,44,45]. Islam et al. noted that some dietary phytochemicals, such as epigallocatechin gallate (EGCG), curcumin, isoliquiritigenin, genistein, and resveratrol, may reduce the expression of inflammatory cytokines in fibroids, i.e., inter alia, interleukines: IL1β, IL6, IL8, IL10, IL12, IL13, TNFα, prostaglandin E2 (PGE-2), and other inflammatory mediators such as cyclooxygenase-2 (COX-2) and nitric oxide (NO). Dietary phytochemicals, by inhibiting the secretion of VEGF, bFGF, PDGF, and hypoxia-inducible factor 1 (HIF-1), may also suppress angiogenesis [27]. It was emphasized that vitamin D can act as an inflammatory suppressant by diminishing the production of pro-inflammatory cytokines, including TNFα and IL6, in myoma. [44]. Our study results demonstrated significant reduction in the expression of inflammatory cytokines IL6 and IL10, influenced by UPA therapy, when a substantial decrease in myoma volume after treatment was observed. The decrease of these cytokines was insignificant or not even noticeable in less or non-responsive fibroids to UPA treatment. This may suggest that, contrary to some studies [46,47], IL10 has pro-inflammatory properties in uterine myomas; hence, the anti-inflammatory effect of UPA can be confirmed.

Other inflammatory mediators, that is, TNFα or TGFβ3 serum levels, were also assessed as possible markers of UFs occurrence [26]. Data concerning TNFα concentration in the UPA-treated fibroids is ambiguous. Some researchers show a significant reduction in TNFα levels [48]. Our study, similarly to Ciebiera et al. [49], does not confirm these findings. To the contrary, we noted no UPA-mediated TNFα response in myoma tissue, regardless of the level of response to the treatment. Progesterone influenced increase in TGFβ with simultaneous decrease in TNFα levels was noted by some studies [45], yet not confirmed by others [50]. TGFβ was suggested to be a key player in the accumulation of excessive ECM in leiomyoma [10]. However, data on the expression of TGFβ in fibroids remain the subject of an academic dispute. Faraji et al. revealed that more than 60% of leiomyoma did not express TGFβ1, higher levels of which can be found in normal myometrium [51]. In contrast, other researchers observed higher levels of TGFβ3 in myoma tissue and emphasized the importance of this growth factor in the proliferation of ECM [52]. More recent studies demonstrated effects of TGFβ and highlighted its increased synthesis and release in uterine fibroids [53]. In UPA-treated myomas, in comparison with the untreated ones, a significantly lower TGFβ3 concentration was shown by other studies, pointing towards the fact that this could be a possible inhibitory mechanism of UPA action in fibroids [35,48,54]. Our results noted significantly lower TGFβ1 concentrations in groups with a good response to the UPA therapy. A similar reaction was not observed in fibroids that, in terms of volume reduction, had responded to the UPA treatment weakly or not at all. These findings complement our previous research [29]. The significant shrinkage of fibroids observed in groups responding well to the UPA treatment may thus have been possible as a result of a decrease in TGFβ expression. Greater amount of fibrosis and simultaneously increased TGFβ expression were noted in myomas with weak and no response to treatment.

In 2017, Demura et al. reported UPA-induced reduction in angiogenesis [31], namely a reduction in VEGF and TGFβ expression. In the same manenr, Yoshida et al. observed suppressed expression of growth factors such as EGF, insulin-like growth factor 1 (IGF-1), TGFβ, and also angiogenic factors (VEGF, ADM) and their receptors in leiomyoma cells influenced by asoprisnil and CDB-2914 (ulipristal acetate) [55]. Xu et al. [23] noted that, by reducing VEGF as well as ADM and its receptor content in fibroid cells, CDB-2914 inhibits growth and angiogenesis. The results were obtained in vitro in 2D cultured purified cells. Some researchers indicate a 3D co-cultured cell model as a preferred study model for vascularization assessment and a more promising predictor of fibroids’ response to therapy. Using 3D leiomyoma cell cultures, Malik et al. demonstrated the mechanism of aberrant ECM formation and drug effectiveness [56]

Many studies indicate that various endothelial markers may be used for vascularization evaluation; hence, CD31 was used as a vessel marker in this study. Assays in leiomyoma tumors (measured using vWF, CD34, and CD31) revealed lower microvessel density in tumors than in surrounding myometrium [57,58,59], especially in smaller fibroids. Some authors have observed a reduction in the numbers of vessels in response to the GnRHa treatment of myomas [15,60]; others have seen no difference in microvessel counts after GnRHa therapy [61]. In our research, we demonstrated that, regardless of the clinical response to treatment (short-term 5 mg UPA), microvessel density, the mean vessel area, and mean vessel perimeter did not differ significantly in non-treated and all UPA-treated groups.

It has to be noted that in specimens retrieved from large myomas (>10 cm), a region adjacent to the tumor periphery is the most biologically active zone. It is characterized by higher levels of gene expression, a higher density of vessels, a higher proliferative rate, and a lower level of hyaline degeneration. [62]. Fibroids of this size were not the subject of our study, the largest being only 7.5 cm in diameter.

In 2020, the European Commission issued the final and legally binding decision concerning the use of UPA for treating uterine fibroids. A restricted implementation of UPA-containing medicines was recommended and limited them only to the treatment of fibroids in premenopausal women with contraindications for surgery (including embolization) [63].

The assessment of mediators of inflammation and angiogenesis, and their comparison in groups of myomas according to their response to the UPA treatment is the strength of the study, as it complements the existing knowledge on evaluating mechanisms of SPRM action in myoma tissue. The evaluation of the parameters studied in relation to the in vivo response to treatment may represent an original approach to the study of the mechanism of drug action. The relatively small amount of assessed myoma tissue, especially in the group of tumors non-responsive to UPA (volume shrinkage), may pose limitations to the study. The pattern of angiogenic factors in fibroids may constitute another study drawback: for instance, VEGF expression steadily increases in VEGF outwardly from the central zone to the periphery of the fibroid. Due to different surgical protocols, it was assumed that the largest fibroids will be selected for examination. In the case of laparoscopic amputation of the uterine body and the need for morcellation, it would not be feasible to identify the exact fibroids with the best response to treatment. All myomas were histologically evaluated after the surgery and no significant morphological differences were found between them. Given the number of planned immunohistochemical reactions, we decided that the most reproducible results for each patient could be obtained by examining the largest tumors. We are aware that this decision can limit the findings.

Taking into account relatively low numbers of assessed myomas, especially those non-responsive to the UPA treatment, the above conclusions should be approached with caution.

## 5. Conclusions

The results of the study indicate that a good response to UPA, manifested by the volume reduction of myoma, may be associated with a decrease in inflammatory mediators and inflammatory cells, but it has no influence on myoma vascularization. The decrease of TGFβ, IL6, and IL10 immunoexpression, as well as the macrophage and mast cell infiltration in fibroids, observed in cases of a good response to the UPA treatment may be an important component of the total effect of UPA on uterine fibroid tissue. Further research is necessary, however.

## Figures and Tables

**Table 1:** Demographic and clinical characteristic of the groups.

Group	Study Markers Range X ± SD							
	Age *	Number of Fibroids *	Days between End of Therapy and Surgery	The Volume of Fibroids before Treatment [cm^3^] *	The Volume of Fibroids after Treatment [cm^3^] *	Change in the Volume after Treatment [%]	Total Cell Density [Number of Cells/1 mm^2^]	Type of Surgery *
Control (*n* = 30)	33–51 41.8 ± 4.6	1–4 1.6 ± 0.8	−	5.5–267.8 76.4 ± 68.2	-	-	130.3–5521.9 1796.8 ± 1964.2	M/ASH/LSH
Good response (*n* = 20)	33–55 42.1 ± 5.3	1–4 1.8 ± 0.8	1–7 3.5 ± 1.7	16.0–297.8 81.5 ± 71.7	0.1–124.2 46.3 ^a^ ± 41.8	−25.4–99.6 −58.6 ± 20.5	4096.5–5415.8 4841.9 ^b^ ± 489.5	M/ASH/LSH
Weak response (*n* = 10)	33–47 43.3 ± 5.4	1–3 1.7 ± 0.9	1–7 3.1 ± 1.9	2.9–199.9 38.0 ± 61.3	1.2–191.8 33.7 ± 59.0	−4.0–23.3 −15.6 ± 6.4	1725.3–3642.6 2551.0 ± 707.7	M/ASH/LSH
No response (*n* = 4)	41–45 43.0 ± 1.6	1–2 1.5 ± 0.6	2–5 3.0 ± 1.4	18.1–68.4 45.9 ± 20.8	25.9–94.1 67.3 ± 29.4	+1.6–104.7 +51.5 ± 42.5	1535.1–1947.1 1702.6 ± 174.1	M/LSH

^a^*p* < 0.05 vs. volume before treatment (Mann–Whitney U test); ^b^ *p* < 0.05 vs. control (Kruscall–Wallis test); and * data regarding good, weak, and no reaction to treatment were previously published by Szydłowska et al. (2021). Abbreviations: ASH—abdominal supracervical hysterectomy; LSH—laparoscopic supracervical hysterectomy, M—Myomectomy; and X ± SD—arithmetical mean ± standard deviation.

**Table 2:** The percentage of TGFβ 1, TNFα, IL6, IL10, CD117, and CD68-positive cells in fibroids in the control group and study groups after 3 months of treatment with ulipristal acetate (UPA).

Group	Study Markers Median (Range) X ± SD					
	TGFβ 1	TNFα	IL6	IL10	CD117	CD68
Control (*n* = 30)	27.7 (21.0–38.6) 28.9 ± 4.9	35.8 (26.7–43.9) 35.7 ± 4.7	39.2 (28.9–53.9) 39.9 ± 5.4	27.5 (18.3–40.8) 28.5 ± 5.9	0.8 (0.4–1.8) 0.9 ± 0.4	3.6 (1.0–5.2) 3.4 ± 1.1
Good response (*n* = 20)	20.5 ^a^ (8.4–43.0) 21.7 ± 8.7	33.3 (23.9–37.4) 32.8 ± 3.8	31.6 ^a^ (23.7–44.3) 33.5 ± 5.8	19.9 ^a,b,c^ (4.2–27.2) 18.0 ± 7.9	0.5 ^a^ (0.2–0.9) 0.5 ± 0.2	1.3 ^a,c^ (0.6–4.7) 1.6 ± 1.0
Weak response (*n* = 10)	25.5 (16.1–31.2) 24.7 ± 4.6	33.5 (27.6–42.3) 34.4 ± 5.4	37.2 (31.5–51.5) 38.8 ± 6.5	27.4 (19.3–35.6) 27.9 ± 4.5	0.7 (0.2–1.0) 0.6 ± 0.3	3.0 (1.5–5.6) 3.1 ± 1.2
No response (*n* = 4)	26.6 (23.9–35.2) 28.1 ± 4.9	34.9 (33.1–37.7) 35.2 ± 2.1	39.3 (34.8–44.4) 39.5 ± 3.9	27.8 (27.7–29.3) 28.2 ± 0.8	0.6 (0.4–2.3) 1.0 ± 0.9	3.5 (3.0–5.1) 3.8 ± 1.0

Note: X ± SD—arithmetical mean ± standard deviation; CD68—marker of cells of the macrophage lineage; CD117—marker of mast cells; IL6—interleukin 6; IL10—interleukin 10; TGFβ 1—transforming growth factor β 1; TNFα—tumor necrosis factor α; ^a^—*p* < 0.001 vs. control group; ^b^—*p* < 0.05 vs. group with a weak response; and ^c^—*p* < 0.05 vs. group with no response to UPA treatment (Kruscall–Wallis test).

**Table 3:** Vessel parameters (based on CD31-positive endothelial cells) in fibroids in the control group and in the study groups after 3 months of treatment with ulipristal acetate (UPA): good, weak, and no response to treatment.

Group	Vessel Parameters Median (Range) X ± SD		
	Vessel Density	Mean Vessel Area (µm^2^)	Mean Vessel Perimeter (µm)
Control (*n* = 30)	2.5 × 10^−5^ (3.9 × 10^−6^–9.7 × 10^−5^) 3.2 × 10^−5^ ± 2.9 × 10^−5^	276.2 (220.0–342.2) 277.6 ± 32.9	89.9 (71.0–99.5) 89.8 ± 6.3
Good response (*n* = 20)	2.2 × 10^−5^ (1.0 × 10^−6^–8.5 × 10^−5^) 2.7 × 10^−5^ ± 2.0 × 10^−5^	296.0 (241.2–341.7) 295.6 ± 28.7	89.0 (60.9–96.1) 84.1 ± 11.7
Weak response (*n* = 10)	3.6 × 10^−5^ (1.3 × 10^−5^–1.0 × 10^−3^) 1.4 × 10^−4^ ± 3.0 × 10^−4^	289.4 (263.2–350.7) 294.8 ± 29.5	87.1 (79.6–96.6) 87.6 ± 5.2
No response (*n* = 4)	7.3 × 10^−5^ (2.3 × 10^−5^–9.3 × 10^−5^) 6.6 × 10^−5^ ± 3.1 × 10^−5^	288.3 (262.6–351.4) 297.7 ± 42.6	90.7 (85.1–94.5) 90.2 ± 3.9

Note: X ± SD—arithmetical mean ± standard deviation and microvessel density = number of vessels per unit area (µm^2^).

## Data Availability

The data presented in this study are available upon the reasonable request from the corresponding author.

## Supplementary Materials

The following are available online at www.mdpi.com/article/10.3390/jcm10163721/s1, Figure S1: The percentage of TGFβ 1-, TNFα-, IL6-, IL10-, CD117- and CD68-positive cells in fibroids in the control group and in the study groups after 3 months of treatment with ulipristal acetate (UPA).
