# Supplementary figures and images for: Markers of Inflammation and Vascular Parameters in Selective Progesterone Receptor Modulator (Ulipristal Acetate)-Treated Uterine Fibroids
Source: J Clin Med. 2021 Aug 21;10(16):3721. doi: 10.3390/jcm10163721 (PMC8397116; doi:10.3390/jcm10163721)

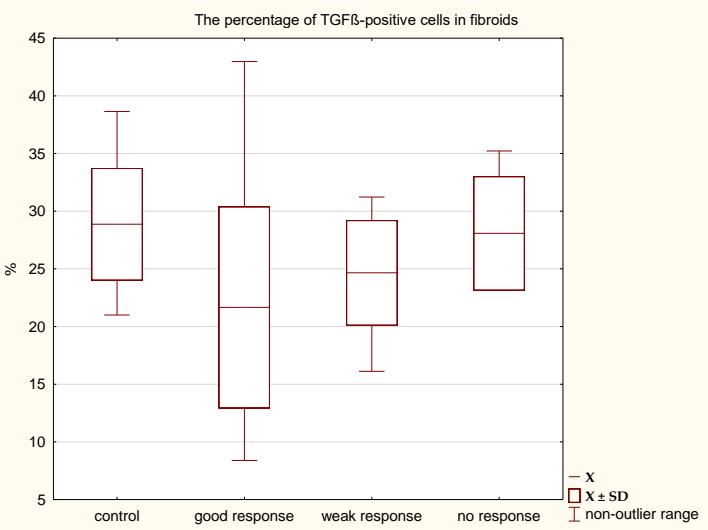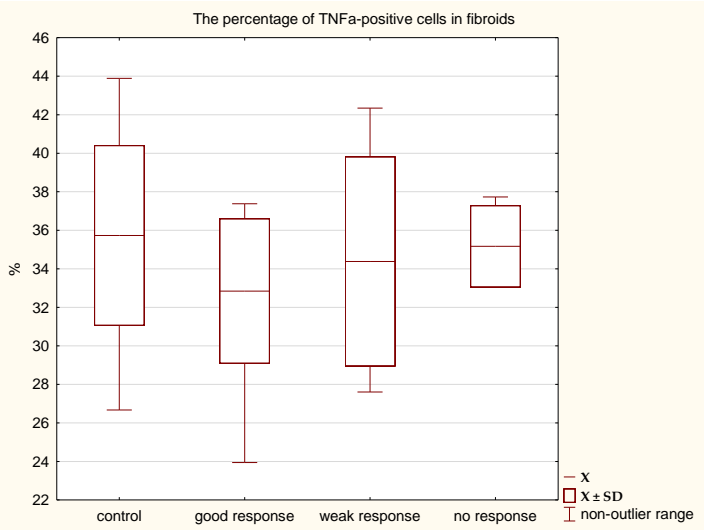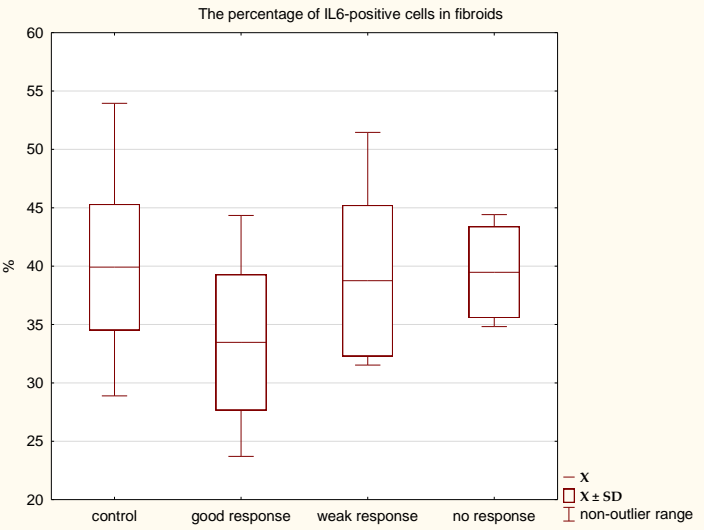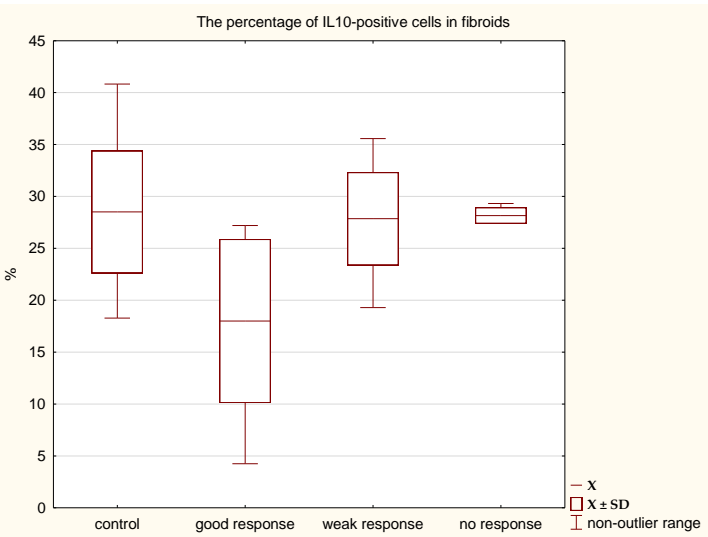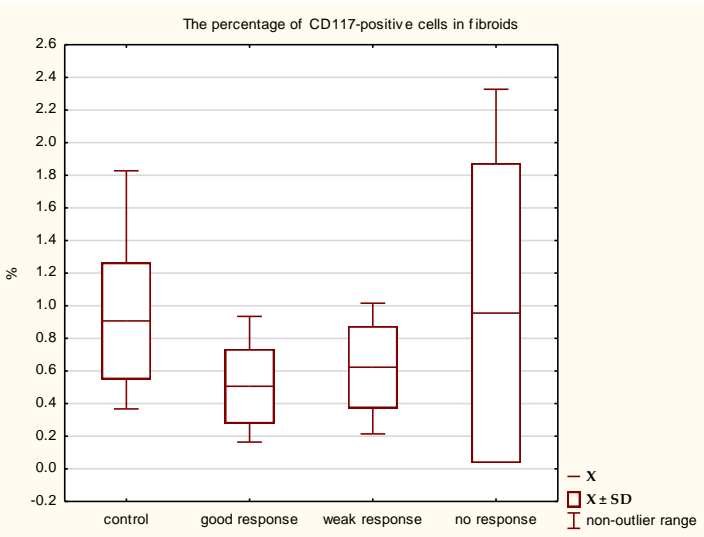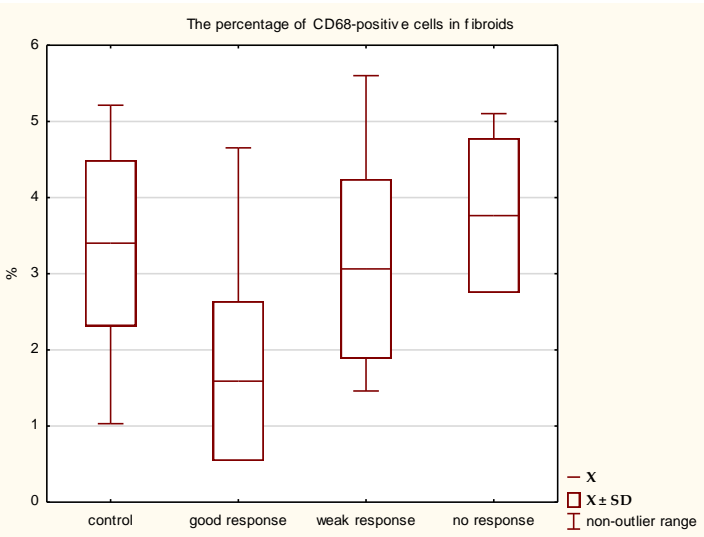

Supplement: Supplementary file 1 [file jcm-10-03721-s001.zip › jcm-1294739-supplementary.pdf]
